# (*E*)-3-(2-Chloro­phen­yl)-1-(4,4′′-difluoro-5′-meth­oxy-1,1′:3′,1′′-terphenyl-4′-yl)prop-2-en-1-one

**DOI:** 10.1107/S1600536812017692

**Published:** 2012-04-28

**Authors:** S. Samshuddin, Badiadka Narayana, Hemmige S. Yathirajan, Richard Betz, Thomas Gerber, Eric Hosten

**Affiliations:** a Mangalore University, Department of Studies in Chemistry, Mangalagangotri 574 199, India; b University of Mysore, Department of Studies in Chemistry, Manasagangotri, Mysore 570 006, India; c Nelson Mandela Metropolitan University, Summerstrand Campus, Department of Chemistry, University Way, Summerstrand, PO Box 77000, Port Elizabeth 6031, South Africa

## Abstract

The title compound, C_28_H_19_ClF_2_O_2_, is a polysubstituted terphenyl derivative bearing a Michael system in which the C=C double bond has an *E* conformation. In the crystal, C—H⋯Cl and C—H⋯O contacts connect the mol­ecules into layers lying perpendicular to the *a* axis. The shortest inter­centroid distance between symmetry-related 4-fluoro­phenyl groups is 3.7547 (16) Å.

## Related literature

For pharmacological background information about terphen­yls, see: Astrue (2002 ▶); Liu (2006 ▶). For the crystal structures of other terphenyl derivatives, see: Betz *et al.* (2011*a* ▶,*b* ▶,*c* ▶,*d* ▶,*e* ▶); Samshuddin *et al.* (2011 ▶). For graph-set analysis, see: Etter *et al.* (1990 ▶); Bernstein *et al.* (1995 ▶).
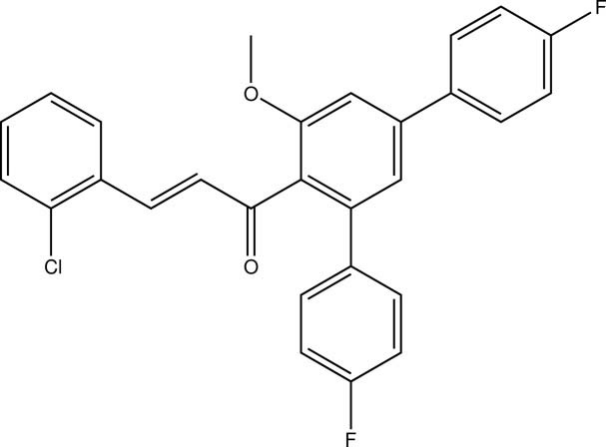


## Experimental

### 

#### Crystal data

C_28_H_19_ClF_2_O_2_
*M* *_r_* = 460.88
Monoclinic, 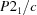

*a* = 14.2065 (7) Å
*b* = 6.8651 (3) Å
*c* = 22.4817 (11) Å
β = 101.406 (2)°
*V* = 2149.32 (18) Å^3^
*Z* = 4
Mo *K*α radiation
μ = 0.22 mm^−1^
*T* = 200 K
0.40 × 0.20 × 0.18 mm

#### Data collection

Bruker APEXII CCD diffractometer
Absorption correction: multi-scan (*SADABS*; Bruker, 2008 ▶) *T*_min_ = 0.918, *T*_max_ = 0.962
17828 measured reflections
5318 independent reflections
3817 reflections with *I* > 2σ(*I*)
*R*_int_ = 0.051

#### Refinement

*R*[*F* ^2^ > 2σ(*F* ^2^)] = 0.064
*wR*(*F* ^2^) = 0.141
*S* = 1.07
5318 reflections
299 parameters
H-atom parameters constrained
Δρ_max_ = 0.41 e Å^−3^
Δρ_min_ = −0.31 e Å^−3^

### 

Data collection: *APEX2* (Bruker, 2010 ▶); cell refinement: *SAINT* (Bruker, 2010 ▶); data reduction: *SAINT*; program(s) used to solve structure: *SHELXS97* (Sheldrick, 2008 ▶); program(s) used to refine structure: *SHELXL97* (Sheldrick, 2008 ▶); molecular graphics: *ORTEP-3* (Farrugia, 1997 ▶) and *Mercury* (Macrae *et al.*, 2008 ▶); software used to prepare material for publication: *SHELXL97* and *PLATON* (Spek, 2009 ▶).

## Supplementary Material

Crystal structure: contains datablock(s) I, global. DOI: 10.1107/S1600536812017692/su2410sup1.cif

Supplementary material file. DOI: 10.1107/S1600536812017692/su2410Isup2.cdx

Structure factors: contains datablock(s) I. DOI: 10.1107/S1600536812017692/su2410Isup3.hkl

Supplementary material file. DOI: 10.1107/S1600536812017692/su2410Isup4.cml

Additional supplementary materials:  crystallographic information; 3D view; checkCIF report

## Figures and Tables

**Table 1:** Hydrogen-bond geometry (Å, °)

*D*—H⋯*A*	*D*—H	H⋯*A*	*D*⋯*A*	*D*—H⋯*A*
C23—H23⋯Cl1^i^	0.95	2.73	3.641 (2)	161
C33—H33⋯Cl1^ii^	0.95	2.76	3.697 (3)	170
C43—H43⋯O1^i^	0.95	2.54	3.411 (3)	153

Symmetry codes: (i) 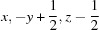; (ii) 
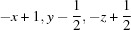
.

**Fig. 1.**
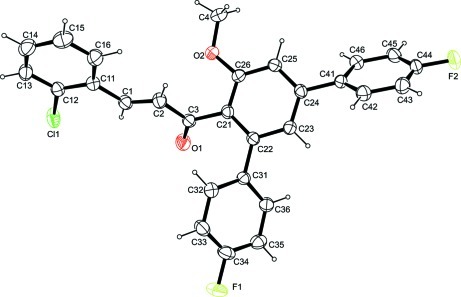
The molecular structure of the title molecule, with atom numbering. Displacement ellipsoids are drawn at the 50% probability level.

**Fig. 2.**
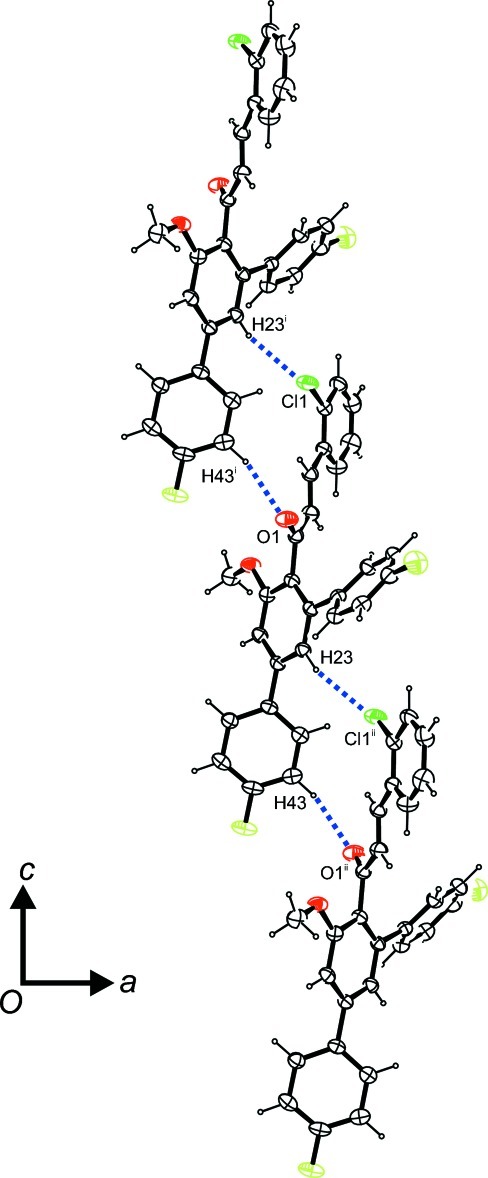
A view along b axis of the C–H···O and C–H···Cl contacts (dashed lines) in the crystal structure of the title compound [symmetry operators: (i) x, -y + 1/2, z + 1/2; (ii) x, -y + 1/2, z - 1/2].

**Fig. 3.**
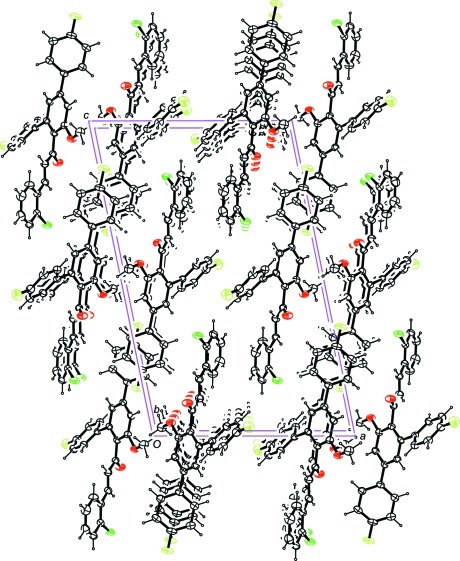
Molecular packing of the title compound, viewed along the b axis (displacement ellipsoids are drawn at the 50% probability level).

## supplementary crystallographic information

### Comment

Polysubstituted aromatics are key structures of great efficacy in synthetic, medicinal and natural product chemistry. Terphenyl derivatives exhibit a considerable range of biological activities and show anticoagulant, immunosuppressant, antithrombotic, neuroprotective, specific 5-lipoxygenase inhibitory and cytotoxic activity effects (Liu, 2006). Due to their promising biological activities terphenyls have received increasing research interest. Therefore, synthesis of polysubstituted aromatics has been a fascinating area in the field of organic chemistry (Astrue, 2002). The molecular and crystal structures of several terphenyl derivatives (Samshuddin *et al.*, 2011; Betz *et al.*, 2011*a*,*b*,*c*,*d*,*e*) have already been reported. In view of the importance of these derivatives, the title compound was prepared and its molecular and crystal structure is reported.

The C═C double of the Michael system has an *E* conformation. The mean planes of the *para*-fluoro phenyl rings, (C31-C36) and (C41-C46), of the terphenyl moiety and the central phenyl ring (C21-C26), enclose angles of 43.39 (12)° and 49.65 (13)°, respectively (Fig. 1).

In the crystal, two different C–H···Cl contacts whose range falls by more than 0.1 Å below the sum of van-der-Waals radii of the atoms participating are observed (Fig. 2 and Table 1). These are supported by two different hydrogen atoms of the terphenyl moiety. Apart from these, a C–H···O contact involving a hydrogen atom from one of the *para*-fluoro phenyl ring (C41-C46) and the ketonic oxygen atom O1 is apparent (Fig. 2 and Table 1). In terms of graph-set analysis (Etter *et al.*, 1990; Bernstein *et al.*, 1995), the descriptor for the C–H···F contacts is *C*^1^_1_(10)*C*^1^_1_(12) on the unary level while the C–H···O contacts necessitate a *C*^1^_1_(10) descriptor on the same level. In total, the molecules are connected to form layers lying perpendicular to the *a* axis (Fig. 3).

The shortest intercentroid distance between two π systems was found at 3.7547 (16) Å and is apparent between the *para*-fluoro phenyl ring (C41-C46) and its symmetry-generated (-x, y-1/2, -z-1/2) equivalent.

### Experimental

To a mixture of 1-(4,4''-difluoro-5'-methoxy-1,1':3',1''-terphenyl-4'-yl) ethanone (0.338 g, 0.001 mol) and 2-chlorobenzaldehyde (0.104 g, 0.001 mol) in 30 ml of ethanol, 1 ml of a 10% sodium hydroxide solution was added and stirred at 278–283 K for 3 h. The precipitate formed was collected by filtration and purified by recrystallization from ethanol. Single crystals were grown from DMF by slow evaporation at room temperature. The yield of the title compound was 81% (m.p.: 452 K).

### Refinement

Carbon-bound H atoms were placed in calculated positions and were included in the refinement in the riding model approximation: C—H = 0.95 Å for aromatic and vinylic H atoms and 0.98 Å for methyl H atoms, with U_iso_(H) = k × U_eq_(C), where k = 1.5 for methyl H atoms and = 1.2 for other H atoms. The CH_3_ H atoms were allowed to rotate with a fixed angle around the C—C bond to best fit the experimental electron density (HFIX 137 in the SHELXL; Sheldrick, 2008).

### Crystal data

**Table:**

C_28_H_19_ClF_2_O_2_	*F*(000) = 952
*M**_r_* = 460.88	*D*_x_ = 1.424 Mg m^−^^3^
Monoclinic, *P*2_1_/*c*	Melting point: 452 K
Hall symbol: -P 2ybc	Mo *K*α radiation, λ = 0.71073 Å
*a* = 14.2065 (7) Å	Cell parameters from 7266 reflections
*b* = 6.8651 (3) Å	θ = 2.6–28.2°
*c* = 22.4817 (11) Å	µ = 0.22 mm^−^^1^
β = 101.406 (2)°	*T* = 200 K
*V* = 2149.32 (18) Å^3^	Block, yellow
*Z* = 4	0.40 × 0.20 × 0.18 mm

### Data collection

**Table:**

Bruker APEXII CCD diffractometer	5318 independent reflections
Radiation source: fine-focus sealed tube	3817 reflections with *I* > 2σ(*I*)
Graphite monochromator	*R*_int_ = 0.051
φ and ω scans	θ_max_ = 28.4°, θ_min_ = 1.9°
Absorption correction: multi-scan (*SADABS*; Bruker, 2008)	*h* = −18→18
*T*_min_ = 0.918, *T*_max_ = 0.962	*k* = −9→8
17828 measured reflections	*l* = −29→29

### Refinement

**Table:**

Refinement on *F*^2^	Primary atom site location: structure-invariant direct methods
Least-squares matrix: full	Secondary atom site location: difference Fourier map
*R*[*F*^2^ > 2σ(*F*^2^)] = 0.064	Hydrogen site location: inferred from neighbouring sites
*wR*(*F*^2^) = 0.141	H-atom parameters constrained
*S* = 1.07	*w* = 1/[σ^2^(*F*_o_^2^) + (0.0356*P*)^2^ + 2.7063*P*] where *P* = (*F*_o_^2^ + 2*F*_c_^2^)/3
5318 reflections	(Δ/σ)_max_ < 0.001
299 parameters	Δρ_max_ = 0.41 e Å^−^^3^
0 restraints	Δρ_min_ = −0.31 e Å^−^^3^

### Fractional atomic coordinates and isotropic or equivalent isotropic displacement parameters (Å^2^)

**Table:**

	*x*	*y*	*z*	*U*_iso_*/*U*_eq_	
Cl1	0.32945 (5)	0.46688 (11)	0.32261 (3)	0.0424 (2)	
F1	0.48872 (13)	−0.3590 (3)	0.05070 (8)	0.0542 (5)	
F2	−0.02840 (13)	0.4610 (3)	−0.34871 (6)	0.0483 (4)	
O1	0.21690 (15)	0.2023 (3)	0.11598 (8)	0.0394 (5)	
O2	0.10962 (14)	0.6409 (3)	0.04792 (7)	0.0360 (4)	
C1	0.29266 (17)	0.5306 (4)	0.18625 (10)	0.0286 (5)	
H1	0.2750	0.4189	0.2065	0.034*	
C2	0.27497 (18)	0.5259 (4)	0.12635 (10)	0.0308 (5)	
H2	0.2916	0.6354	0.1048	0.037*	
C3	0.22992 (17)	0.3548 (4)	0.09182 (10)	0.0266 (5)	
C4	0.0579 (2)	0.8171 (4)	0.03123 (12)	0.0380 (6)	
H4A	0.0929	0.8975	0.0068	0.057*	
H4B	0.0512	0.8885	0.0679	0.057*	
H4C	−0.0059	0.7863	0.0075	0.057*	
C11	0.33711 (17)	0.6927 (4)	0.22443 (10)	0.0280 (5)	
C12	0.35889 (17)	0.6785 (4)	0.28769 (10)	0.0283 (5)	
C13	0.40353 (19)	0.8263 (4)	0.32436 (12)	0.0353 (6)	
H13	0.4168	0.8114	0.3672	0.042*	
C14	0.4286 (2)	0.9946 (4)	0.29868 (13)	0.0410 (7)	
H14	0.4612	1.0954	0.3235	0.049*	
C15	0.4060 (2)	1.0165 (4)	0.23610 (14)	0.0440 (7)	
H15	0.4217	1.1341	0.2180	0.053*	
C16	0.3608 (2)	0.8686 (4)	0.20019 (12)	0.0378 (6)	
H16	0.3453	0.8869	0.1575	0.045*	
C21	0.19968 (16)	0.3801 (4)	0.02426 (10)	0.0250 (5)	
C22	0.22967 (16)	0.2553 (3)	−0.01771 (10)	0.0236 (5)	
C23	0.19342 (17)	0.2854 (4)	−0.07951 (10)	0.0262 (5)	
H23	0.2142	0.2030	−0.1083	0.031*	
C24	0.12777 (16)	0.4327 (4)	−0.09994 (10)	0.0250 (5)	
C25	0.09855 (17)	0.5559 (4)	−0.05830 (10)	0.0271 (5)	
H25	0.0537	0.6570	−0.0716	0.033*	
C26	0.13545 (17)	0.5300 (4)	0.00332 (10)	0.0261 (5)	
C31	0.29922 (17)	0.0940 (3)	0.00053 (10)	0.0245 (5)	
C32	0.38279 (17)	0.1197 (4)	0.04463 (11)	0.0301 (5)	
H32	0.3961	0.2434	0.0634	0.036*	
C33	0.44637 (19)	−0.0325 (4)	0.06131 (11)	0.0359 (6)	
H33	0.5026	−0.0150	0.0916	0.043*	
C34	0.4265 (2)	−0.2090 (4)	0.03319 (12)	0.0360 (6)	
C35	0.34726 (19)	−0.2412 (4)	−0.01133 (11)	0.0332 (6)	
H35	0.3361	−0.3644	−0.0307	0.040*	
C36	0.28382 (18)	−0.0874 (4)	−0.02719 (11)	0.0294 (5)	
H36	0.2282	−0.1067	−0.0579	0.035*	
C41	0.08695 (17)	0.4497 (4)	−0.16619 (10)	0.0263 (5)	
C42	0.14603 (19)	0.4464 (4)	−0.20846 (11)	0.0314 (5)	
H42	0.2137	0.4398	−0.1949	0.038*	
C43	0.1081 (2)	0.4526 (4)	−0.27020 (11)	0.0350 (6)	
H43	0.1487	0.4518	−0.2991	0.042*	
C44	0.0103 (2)	0.4598 (4)	−0.28813 (10)	0.0335 (6)	
C45	−0.05083 (19)	0.4642 (4)	−0.24832 (11)	0.0342 (6)	
H45	−0.1184	0.4694	−0.2625	0.041*	
C46	−0.01178 (18)	0.4607 (4)	−0.18650 (11)	0.0300 (5)	
H46	−0.0529	0.4659	−0.1580	0.036*	

### Atomic displacement parameters (Å^2^)

**Table:**

	*U*^11^	*U*^22^	*U*^33^	*U*^12^	*U*^13^	*U*^23^
Cl1	0.0517 (4)	0.0472 (4)	0.0245 (3)	−0.0136 (3)	−0.0019 (3)	0.0057 (3)
F1	0.0605 (11)	0.0418 (10)	0.0580 (11)	0.0263 (9)	0.0060 (9)	0.0116 (8)
F2	0.0676 (12)	0.0481 (10)	0.0226 (7)	0.0027 (9)	−0.0072 (7)	0.0013 (7)
O1	0.0562 (12)	0.0328 (11)	0.0284 (9)	−0.0035 (10)	0.0062 (8)	0.0048 (8)
O2	0.0450 (11)	0.0365 (11)	0.0255 (8)	0.0141 (9)	0.0047 (7)	−0.0039 (8)
C1	0.0295 (12)	0.0278 (13)	0.0275 (11)	0.0002 (11)	0.0030 (9)	0.0031 (10)
C2	0.0332 (13)	0.0316 (14)	0.0269 (11)	−0.0026 (12)	0.0039 (10)	0.0013 (10)
C3	0.0270 (12)	0.0284 (13)	0.0246 (11)	0.0035 (11)	0.0055 (9)	0.0019 (10)
C4	0.0452 (16)	0.0288 (15)	0.0402 (14)	0.0087 (13)	0.0093 (12)	−0.0050 (11)
C11	0.0282 (12)	0.0287 (13)	0.0264 (11)	0.0008 (11)	0.0032 (9)	−0.0009 (10)
C12	0.0254 (12)	0.0296 (14)	0.0286 (12)	0.0000 (10)	0.0021 (9)	−0.0003 (10)
C13	0.0330 (13)	0.0405 (16)	0.0299 (12)	0.0000 (12)	0.0003 (10)	−0.0079 (11)
C14	0.0370 (15)	0.0354 (16)	0.0486 (16)	−0.0036 (13)	0.0032 (12)	−0.0113 (13)
C15	0.0499 (17)	0.0293 (15)	0.0536 (17)	−0.0066 (13)	0.0118 (14)	0.0007 (13)
C16	0.0485 (16)	0.0338 (15)	0.0301 (13)	−0.0005 (13)	0.0053 (11)	0.0015 (11)
C21	0.0257 (11)	0.0252 (12)	0.0227 (10)	−0.0021 (10)	0.0013 (9)	−0.0006 (9)
C22	0.0231 (11)	0.0219 (12)	0.0246 (11)	−0.0024 (10)	0.0014 (9)	0.0005 (9)
C23	0.0262 (12)	0.0264 (13)	0.0249 (11)	0.0006 (10)	0.0027 (9)	−0.0037 (9)
C24	0.0241 (11)	0.0260 (13)	0.0233 (10)	−0.0019 (10)	0.0006 (8)	0.0001 (9)
C25	0.0270 (12)	0.0246 (13)	0.0282 (11)	0.0028 (10)	0.0014 (9)	0.0001 (10)
C26	0.0296 (12)	0.0220 (12)	0.0265 (11)	−0.0005 (10)	0.0049 (9)	−0.0021 (9)
C31	0.0278 (12)	0.0224 (12)	0.0236 (10)	0.0012 (10)	0.0062 (9)	0.0022 (9)
C32	0.0298 (13)	0.0310 (14)	0.0285 (12)	0.0004 (11)	0.0030 (10)	−0.0015 (10)
C33	0.0308 (13)	0.0434 (17)	0.0313 (12)	0.0075 (13)	0.0007 (10)	0.0044 (12)
C34	0.0398 (15)	0.0346 (15)	0.0352 (13)	0.0129 (12)	0.0111 (11)	0.0121 (11)
C35	0.0427 (15)	0.0230 (13)	0.0360 (13)	0.0007 (12)	0.0130 (11)	0.0019 (11)
C36	0.0324 (13)	0.0268 (13)	0.0287 (12)	−0.0006 (11)	0.0052 (10)	0.0007 (10)
C41	0.0309 (12)	0.0226 (12)	0.0231 (10)	0.0011 (10)	−0.0006 (9)	−0.0016 (9)
C42	0.0315 (13)	0.0312 (14)	0.0302 (12)	0.0001 (11)	0.0026 (10)	−0.0007 (11)
C43	0.0454 (16)	0.0324 (15)	0.0277 (12)	−0.0013 (13)	0.0085 (11)	−0.0002 (11)
C44	0.0496 (16)	0.0262 (13)	0.0206 (11)	0.0017 (12)	−0.0034 (10)	0.0007 (10)
C45	0.0342 (13)	0.0310 (14)	0.0321 (12)	0.0026 (12)	−0.0063 (10)	0.0001 (11)
C46	0.0304 (13)	0.0306 (14)	0.0278 (11)	0.0020 (11)	0.0026 (9)	0.0006 (10)

### Geometric parameters (Å, º)

**Table:**

Cl1—C12	1.741 (3)	C22—C31	1.487 (3)
F1—C34	1.364 (3)	C23—C24	1.391 (3)
F2—C44	1.364 (3)	C23—H23	0.9500
O1—C3	1.211 (3)	C24—C25	1.385 (3)
O2—C26	1.366 (3)	C24—C41	1.492 (3)
O2—C4	1.426 (3)	C25—C26	1.392 (3)
C1—C2	1.321 (3)	C25—H25	0.9500
C1—C11	1.470 (3)	C31—C36	1.390 (3)
C1—H1	0.9500	C31—C32	1.399 (3)
C2—C3	1.481 (3)	C32—C33	1.384 (4)
C2—H2	0.9500	C32—H32	0.9500
C3—C21	1.504 (3)	C33—C34	1.370 (4)
C4—H4A	0.9800	C33—H33	0.9500
C4—H4B	0.9800	C34—C35	1.368 (4)
C4—H4C	0.9800	C35—C36	1.388 (4)
C11—C16	1.393 (4)	C35—H35	0.9500
C11—C12	1.398 (3)	C36—H36	0.9500
C12—C13	1.380 (3)	C41—C42	1.387 (3)
C13—C14	1.369 (4)	C41—C46	1.389 (3)
C13—H13	0.9500	C42—C43	1.387 (3)
C14—C15	1.388 (4)	C42—H42	0.9500
C14—H14	0.9500	C43—C44	1.368 (4)
C15—C16	1.375 (4)	C43—H43	0.9500
C15—H15	0.9500	C44—C45	1.365 (4)
C16—H16	0.9500	C45—C46	1.391 (3)
C21—C26	1.394 (3)	C45—H45	0.9500
C21—C22	1.402 (3)	C46—H46	0.9500
C22—C23	1.398 (3)		
			
C26—O2—C4	118.60 (19)	C25—C24—C41	121.2 (2)
C2—C1—C11	126.0 (2)	C23—C24—C41	119.3 (2)
C2—C1—H1	117.0	C24—C25—C26	119.4 (2)
C11—C1—H1	117.0	C24—C25—H25	120.3
C1—C2—C3	121.9 (2)	C26—C25—H25	120.3
C1—C2—H2	119.0	O2—C26—C25	123.8 (2)
C3—C2—H2	119.0	O2—C26—C21	114.6 (2)
O1—C3—C2	122.6 (2)	C25—C26—C21	121.5 (2)
O1—C3—C21	120.9 (2)	C36—C31—C32	117.8 (2)
C2—C3—C21	116.4 (2)	C36—C31—C22	120.4 (2)
O2—C4—H4A	109.5	C32—C31—C22	121.7 (2)
O2—C4—H4B	109.5	C33—C32—C31	120.9 (2)
H4A—C4—H4B	109.5	C33—C32—H32	119.5
O2—C4—H4C	109.5	C31—C32—H32	119.5
H4A—C4—H4C	109.5	C34—C33—C32	118.6 (2)
H4B—C4—H4C	109.5	C34—C33—H33	120.7
C16—C11—C12	115.9 (2)	C32—C33—H33	120.7
C16—C11—C1	122.5 (2)	F1—C34—C35	118.8 (3)
C12—C11—C1	121.6 (2)	F1—C34—C33	118.1 (2)
C13—C12—C11	122.6 (2)	C35—C34—C33	123.1 (2)
C13—C12—Cl1	117.85 (19)	C34—C35—C36	117.6 (2)
C11—C12—Cl1	119.51 (19)	C34—C35—H35	121.2
C14—C13—C12	119.7 (2)	C36—C35—H35	121.2
C14—C13—H13	120.1	C35—C36—C31	122.0 (2)
C12—C13—H13	120.1	C35—C36—H36	119.0
C13—C14—C15	119.4 (3)	C31—C36—H36	119.0
C13—C14—H14	120.3	C42—C41—C46	119.0 (2)
C15—C14—H14	120.3	C42—C41—C24	120.9 (2)
C16—C15—C14	120.2 (3)	C46—C41—C24	120.0 (2)
C16—C15—H15	119.9	C43—C42—C41	121.1 (2)
C14—C15—H15	119.9	C43—C42—H42	119.4
C15—C16—C11	122.1 (2)	C41—C42—H42	119.4
C15—C16—H16	119.0	C44—C43—C42	117.8 (2)
C11—C16—H16	119.0	C44—C43—H43	121.1
C26—C21—C22	119.3 (2)	C42—C43—H43	121.1
C26—C21—C3	117.6 (2)	F2—C44—C45	118.1 (2)
C22—C21—C3	123.0 (2)	F2—C44—C43	118.7 (2)
C23—C22—C21	118.5 (2)	C45—C44—C43	123.2 (2)
C23—C22—C31	118.6 (2)	C44—C45—C46	118.4 (2)
C21—C22—C31	122.9 (2)	C44—C45—H45	120.8
C24—C23—C22	121.8 (2)	C46—C45—H45	120.8
C24—C23—H23	119.1	C41—C46—C45	120.5 (2)
C22—C23—H23	119.1	C41—C46—H46	119.8
C25—C24—C23	119.5 (2)	C45—C46—H46	119.8
			
C11—C1—C2—C3	179.9 (2)	C24—C25—C26—C21	−1.5 (4)
C1—C2—C3—O1	−8.7 (4)	C22—C21—C26—O2	178.9 (2)
C1—C2—C3—C21	170.7 (2)	C3—C21—C26—O2	1.8 (3)
C2—C1—C11—C16	4.0 (4)	C22—C21—C26—C25	1.6 (4)
C2—C1—C11—C12	−175.5 (3)	C3—C21—C26—C25	−175.6 (2)
C16—C11—C12—C13	−1.7 (4)	C23—C22—C31—C36	−43.5 (3)
C1—C11—C12—C13	177.8 (2)	C21—C22—C31—C36	137.3 (2)
C16—C11—C12—Cl1	178.4 (2)	C23—C22—C31—C32	135.0 (2)
C1—C11—C12—Cl1	−2.0 (3)	C21—C22—C31—C32	−44.2 (3)
C11—C12—C13—C14	−0.4 (4)	C36—C31—C32—C33	−1.9 (4)
Cl1—C12—C13—C14	179.5 (2)	C22—C31—C32—C33	179.6 (2)
C12—C13—C14—C15	2.0 (4)	C31—C32—C33—C34	0.8 (4)
C13—C14—C15—C16	−1.6 (4)	C32—C33—C34—F1	−178.9 (2)
C14—C15—C16—C11	−0.7 (5)	C32—C33—C34—C35	1.0 (4)
C12—C11—C16—C15	2.3 (4)	F1—C34—C35—C36	178.4 (2)
C1—C11—C16—C15	−177.3 (3)	C33—C34—C35—C36	−1.5 (4)
O1—C3—C21—C26	122.1 (3)	C34—C35—C36—C31	0.2 (4)
C2—C3—C21—C26	−57.3 (3)	C32—C31—C36—C35	1.4 (4)
O1—C3—C21—C22	−54.9 (3)	C22—C31—C36—C35	180.0 (2)
C2—C3—C21—C22	125.6 (3)	C25—C24—C41—C42	134.0 (3)
C26—C21—C22—C23	−0.3 (3)	C23—C24—C41—C42	−48.6 (3)
C3—C21—C22—C23	176.7 (2)	C25—C24—C41—C46	−48.7 (3)
C26—C21—C22—C31	178.9 (2)	C23—C24—C41—C46	128.6 (3)
C3—C21—C22—C31	−4.1 (4)	C46—C41—C42—C43	−0.4 (4)
C21—C22—C23—C24	−1.1 (4)	C24—C41—C42—C43	176.8 (2)
C31—C22—C23—C24	179.7 (2)	C41—C42—C43—C44	−0.7 (4)
C22—C23—C24—C25	1.3 (4)	C42—C43—C44—F2	−178.4 (2)
C22—C23—C24—C41	−176.1 (2)	C42—C43—C44—C45	1.0 (4)
C23—C24—C25—C26	0.0 (4)	F2—C44—C45—C46	179.3 (2)
C41—C24—C25—C26	177.4 (2)	C43—C44—C45—C46	−0.1 (4)
C4—O2—C26—C25	−13.5 (4)	C42—C41—C46—C45	1.4 (4)
C4—O2—C26—C21	169.2 (2)	C24—C41—C46—C45	−175.9 (2)
C24—C25—C26—O2	−178.5 (2)	C44—C45—C46—C41	−1.1 (4)

### Hydrogen-bond geometry (Å, º)

**Table:**

*D*—H···*A*	*D*—H	H···*A*	*D*···*A*	*D*—H···*A*
C23—H23···Cl1^i^	0.95	2.73	3.641 (2)	161
C33—H33···Cl1^ii^	0.95	2.76	3.697 (3)	170
C43—H43···O1^i^	0.95	2.54	3.411 (3)	153

Symmetry codes: (i) *x*, −*y*+1/2, *z*−1/2; (ii) −*x*+1, *y*−1/2, −*z*+1/2.
